# A Comprehensive Review of Calcium Electroporation—A Novel Cancer Treatment Modality

**DOI:** 10.3390/cancers12020290

**Published:** 2020-01-25

**Authors:** Stine K. Frandsen, Mille Vissing, Julie Gehl

**Affiliations:** 1 Center for Experimental Drug and Gene Electrotransfer (C*EDGE), Department of Clinical Oncology and Palliative Care, Zealand University Hospital, Sygehusvej 10, 4000 Roskilde, Denmark; stfra@regionsjaelland.dk (S.K.F.); emlh@regionsjaelland.dk (M.V.); 2 Department of Clinical Medicine, Faculty of Health and Medical Sciences, University of Copenhagen, Blegdamsvej 3B, 2200 Copenhagen, Denmark

**Keywords:** calcium electroporation, in vitro, in vivo, veterinary study, clinical trial

## Abstract

Calcium electroporation is a potential novel anti-cancer treatment where high calcium concentrations are introduced into cells by electroporation, a method where short, high voltage pulses induce transient permeabilisation of the plasma membrane allowing passage of molecules into the cytosol. Calcium is a tightly regulated, ubiquitous second messenger involved in many cellular processes including cell death. Electroporation increases calcium uptake leading to acute and severe ATP depletion associated with cancer cell death. This comprehensive review describes published data about calcium electroporation applied in vitro, in vivo, and clinically from the first publication in 2012. Calcium electroporation has been shown to be a safe and efficient anti-cancer treatment in clinical studies with cutaneous metastases and recurrent head and neck cancer. Normal cells have been shown to be less affected by calcium electroporation than cancer cells and this difference might be partly induced by differences in membrane repair, expression of calcium transporters, and cellular structural changes. Interestingly, both clinical data and preclinical studies have indicated a systemic immune response induced by calcium electroporation. New cancer treatments are needed, and calcium electroporation represents an inexpensive and efficient treatment with few side effects, that could potentially be used worldwide and for different tumor types.

## 1. Introduction

**Figure 1:**
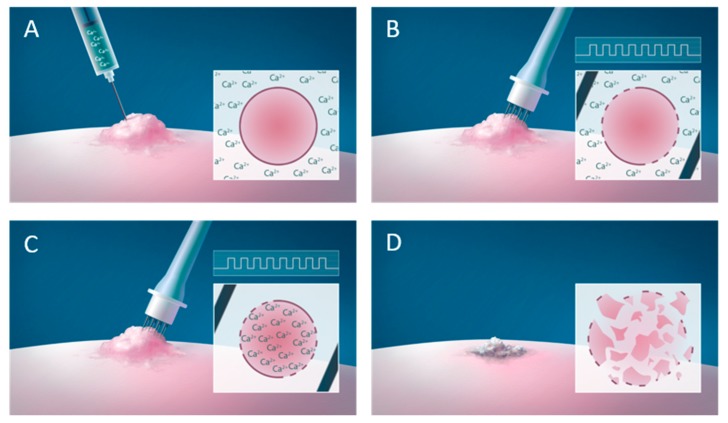
Calcium electroporation. (**A**) Calcium is injected in the tumor causing a high extracellular calcium concentration. (**B**) Immediately after the injection, the tumor is electroporated using an electrode (e.g. needle electrode) causing transient permeabilisation of the cell membrane allowing passage of calcium into the cell (**C**) causing cancer cell death (**D**).

Calcium is a ubiquitous intracellular second messenger involved in many cellular processes including cell death [1,2,3,4,5]. The homeostasis of this tightly regulated ion is severely affected after calcium electroporation, where a high concentration of calcium is introduced into the cell by electroporation, a method where short high-voltage pulses transiently permeabilize the cell membrane allowing increased passage of ions or molecules. This method is used clinically in more than 140 centers in Europe in combination with chemotherapeutic drugs (electrochemotherapy) for anticancer treatment [6,7,8,9,10,11,12,13,14,15,16]. Electroporation increases the uptake and thereby cytotoxicity of the chemotherapeutic drug dramatically [17]. Electroporation in combination with calcium (calcium electroporation, Figure 1) has been tested as a novel anticancer treatment in vitro, in vivo and in clinical trials showing promising effect [18,19].

This review begins with an introduction to calcium homeostasis in normal and malignant cells including a description of calcium signaling, channels, pumps, and mitochondria followed by a short description of electroporation. Then, the effect of calcium electroporation in vitro, in vivo, as well as the proposed cellular and systemic mechanisms of action, are described followed by a description of the veterinary studies and clinical trials. Finally, perspectives of this novel anti-cancer treatment are discussed.

## 2. Normal Cellular Calcium Homeostasis

**Figure 2:**
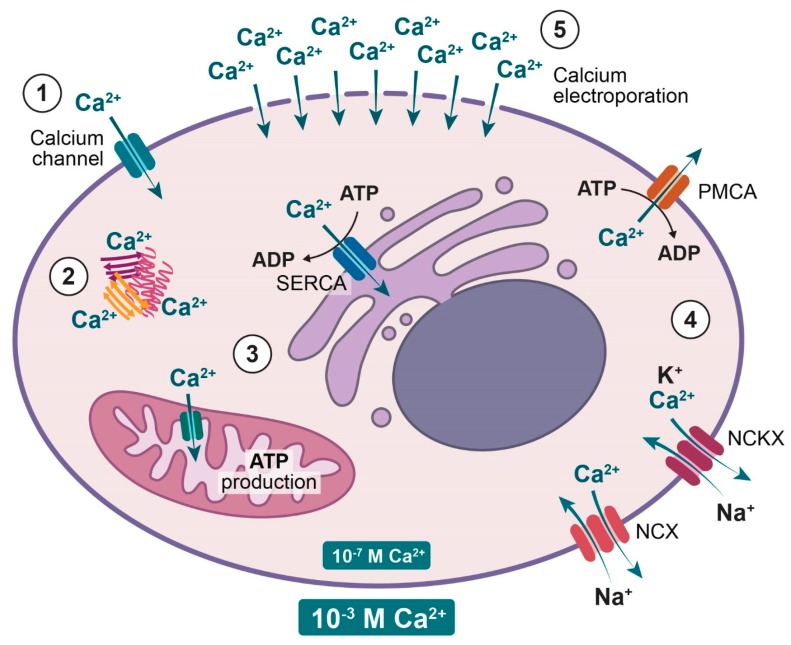
Cellular calcium homeostasis. Calcium is tightly regulated to maintain the low intracellular calcium concentration. (**1**) Calcium can enter the cell through calcium channels. (**2**) Inside the cells calcium is chelated by proteins. (**3**) Mitochondria and endoplasmic reticulum store calcium where transport is facilitated by transporters including the sarco-endoplasmic reticulum calcium ATPase (SERCA). (**4**) Calcium is extruded from the cell by the ATP dependent plasma membrane calcium ATPase (PMCA) and the sodium calcium exchanger (NCX) and the sodium calcium potassium exchanger (NCKX). (**5**) Calcium electroporation induces high intracellular concentrations of calcium by permeabilisation of the plasma membrane in the presence of high extracellular calcium concentrations.

Calcium is an essential messenger involved in numerous intracellular processes from fertilization through development, differentiation, and proliferation to cell death [1,2,5]. There is a 10–20,000 fold concentration gradient of calcium across the plasma membrane; thus, the cell has to chelate calcium by binding to different proteins, compartmentalize into organelles such as endoplasmic reticulum (ER) and mitochondria, or extrude calcium using different pumps (ATPases) and exchangers to maintain ion homeostasis (see Figure 2) [2,20,21]. Inside the cell, calcium cannot diffuse freely and thus acts locally, which gives a non-homogeneous intracellular signal that depends on the shape, location, and duration of the signal [21,22]. Mobile proteins can prolong the calcium signal and increase the area of effect [4] and the calcium signal can propagate through positive feedback processes (Ca^2+^ induced Ca^2+^ release) [22]. 

Calcium is mainly stored in the endoplasmic reticulum (ER), the sarcoplasmic reticulum (SR, in muscle cells) and mitochondria. The sarco-endoplasmic reticulum calcium ATPase (SERCA) pumps calcium into the ER and SR [2,23]. In the mitochondria, calcium ions diffuse freely through pores in the outer membrane but only via ion channels and transporters in the inner membrane [24,25]. Calcium inside the mitochondria can regulate the function, movement, and viability of the organelle. An increased mitochondrial calcium concentration can modulate mitochondrial metabolism by increasing the ATP production but it can also trigger cell death, apoptosis or necrosis, through membrane permeability transition [4,26,27]. Thus, at high intracellular calcium concentrations, as seen after e.g. calcium electroporation, calcium enters the mitochondria and this is likely limiting or destroying the mitochondrial respiration and thereby ATP production.

Calcium is extruded from cells by the ATP-dependent plasma membrane calcium ATPase (PMCA) and the ATP-independent Na^+^/Ca^2+^-exchanger (NCX) and Na^+^/Ca^2+^/K^+^-exchanger (NCKX). PMCA is a calcium pump located in the plasma membrane with high affinity and low capacity and therefore effective at maintaining low intracellular calcium concentration over time [4,28]. NCX and NCKX uses the electrochemical gradient of Na^+^ across the plasma membrane to exchange Ca^2+^ for Na^+^, and Ca^2+^ together with K^+^ for Na^+^, respectively [2,4]. These exchangers with low affinity and high capacity can make rapid adjustments in the intracellular calcium concentration and therefore complement the PMCA for extrusion of calcium out of the cell [21,29]. 

## 3. Normal versus Malignant Calcium Homeostasis

Many of the characteristics that define cancer cells, such as self-sufficiency in growth signals, evasion of apoptosis, insensitivity to anti-growth signals, sustained angiogenesis, limitless replication, and tissue invasion and metastases [30] are regulated by calcium and are often altered in cancer cells compared with normal cells [31,32,33].

Proteins involved in regulating calcium signals, are often remodeled in cancer cells compared to normal cells to sustain proliferation and avoid cell death [32]. Calcium channels, pumps, and exchangers are all present in malignant cells as well as normal cells; however, their expression, localization, and/or activity can be different. A decrease in SERCA2 expression [34,35] and SERCA3 expression [36,37,38] has been observed in several different cancer cell lines and tumor samples, as well as alterations in the genes coding for SERCA2 and SERCA3 have been shown in samples from cancer patients [39,40,41], indicating that transport of calcium from the cytosol to ER could be reduced in cancer cells. Alterations of PMCA have also been shown where an increased expression of PMCA4 has been observed with increasing differentiation of cells, thus lowest PMCA4 expression in cancer cell lines [42,43] and tumors tissues [44,45] compared with normal cells and tissues. However, the opposite has also been observed where PMCA2 expression is up-regulated in breast cancer cell lines [46] and tumor tissue [47] showing the diverse roles of calcium in cell-signaling. 

## 4. Electroporation

As described, calcium is normally a tightly regulated intracellular ion but by using electroporation, supraphysiological concentrations of calcium can be introduced. Electroporation, or electropermeabilisation, is a method where application of short, high-voltage pulses causes transient permeabilisation of the cell membrane allowing passage of otherwise non-permeant ions or molecules into the cell. The method is used in vitro, in vivo, and in the clinic to introduce different ions or molecules, such as chemotherapeutic drugs, genes or calcium, into cells [18,19,48,49,50,51,52,53]. Most clinical trials, and studies with calcium electroporation described in this review, have used eight pulses of 100 μs, 1000 V/cm and 1 Hz. When the membrane is permeabilized by electroporation, water molecules enter the membrane, forming a water bridge, which subsequently leads to hydrophilic pores being formed through which ions and molecules can diffuse [54,55]. 

At a range of electric parameters, electroporation is reversible and the membrane will reseal, diminishing flux of ions and molecules. Resealing is dependent on temperature, degree of permeabilisation, and integrity of the cytoskeleton [51,56]. Cell type also affects resealing, where normal cells seem to repair faster than cancer cells in vitro [57]. Membrane repair is calcium dependent [58,59] and the membrane will fail to repair (and thereby reseal) without calcium present [60,61]. It has also been shown that calcium reduces pore lifetime after electroporation [62] and reduces the area per lipid in the membrane [63]. 

The first clinical trial using reversible electroporation in combination with a chemotherapeutic drug (electrochemotherapy) was performed in 1990–1991 [6]. Since then several clinical trials using electrochemotherapy have been performed for treatment of small tumors, such as cutaneous and subcutaneous metastases [7,8,9,10,14,64,65], for larger tumors such as chest wall breast cancer recurrences [11,66], as well as for treatment of tumors in internal organs and deep seated tumors [67,68,69,70,71]. Electroporation is also investigated for use in gene electrotransfer where it can be used for DNA vaccine [72] and treatment of cancers [73,74]. Thus, electroporation is a well-established clinical method and can easily be tested in combination with new or other drugs.

## 5. Calcium Electroporation

In 2003, it was shown in vitro that Ca^2+^ enters cells after electroporation in a calcium-containing buffer and a high extracellular calcium concentration during electroporation decreased viability [75]. Moreover, in 2011, it was described that calcium electroporation can be used to turn off the transgene expression after gene electrotransfer in muscles [76]. However, it was not until 2012 that calcium electroporation was described as a possible novel anti-cancer treatment [18] followed by several studies including the first clinical trial using calcium electroporation only five years later in 2017 [19]. 

### 5.1. Effect on Cancer Cells In Vitro and In Vivo

Calcium electroporation has been tested in vitro in 18 different cell lines (12 cancer cell lines and six normal cell lines of which five are immortalized cells and one is primary cells) [18,77,78,79,80,81,82,83]. In all tested cell lines, calcium electroporation induced cell death in a dose-dependent manner [18,77,78,80,81] with an IC_50_ ranging from 0.4–5.0 mM calcium (in combination with electroporation) in the studies where IC_50_ was determined [80,81]. Similar effects can be reached with calcium electroporation as with electrochemotherapy using bleomycin, but no synergistic effect is seen when combining calcium and bleomycin before electroporation [84]. There was no effect of treatment with calcium alone in concentrations up to 5 mM [18,77,80,81,82] and even up to 20 mM in the two cell lines where such high concentrations were tested [78]. This is unlike treatment with bleomycin alone where some effect is seen; however, this effect is much less than when combined with electroporation [81,84]. 

Calcium electroporation has been tested in vivo in five human tumors (bladder cancer, breast cancer, colon cancer, small cell lung cancer, and rhabdomyosarcoma) grown subcutaneously on immunocompromised mice [18,61,82], since human tumor models can only be tested in mice with lowered immune effect. Additionally, two murine tumors (colon adenocarcinoma and melanoma) grown on immunocompetent mice [79,81] and immunocompromised mice [79] have also been tested. The calcium electroporation treatment induced tumor cell death in all tested tumors but with difference in sensitivity to treatment. The small cell lung cancer tumors were the most sensitive of the tumors treated on immunocompromised mice with 89% of tumors completely eradicated [61]. However, the colon cancer tumors were more sensitive when treated on immunocompetent mice with complete remission of all treated tumors [79]. A similar effect was not seen when the same tumor type was treated on immunocompromised mice, which indicates an immune response being involved. CaCl_2_ was used in all the in vivo studies testing calcium electroporation but it has been shown in vitro that the effect is independent of the calcium compound [84]. In most of the in vivo studies, 168 mM calcium was injected in a volume equivalent to 50% of tumor volume; however, in one of the studies, different calcium doses have been tested. There seems to be a similar effect of calcium electroporation when treating with calcium concentrations between 100–500 mM (injected in a volume equivalent to 50% of the tumor volume) and with injection volumes from 20% to 80% of the tumor volume (with 168 mM CaCl_2_) [61]. The calcium concentrations used in vivo [18,61] are much higher than the concentrations inducing cell death in vitro [77,80], which is also seen when using bleomycin in combination with electroporation [79,84]. This is likely due to the smaller extracellular volume (ECV) in tissue compared with cells in suspension in vitro, which is also supported by a study on spheroids, a 3D in vitro model [85], where the drug concentrations (calcium or bleomycin) were similar to the in vivo studies and the ECV of spheroids is comparable to the ECV of tissue.

### 5.2. Effect on Normal Cells and Tissues

**Figure 3:**
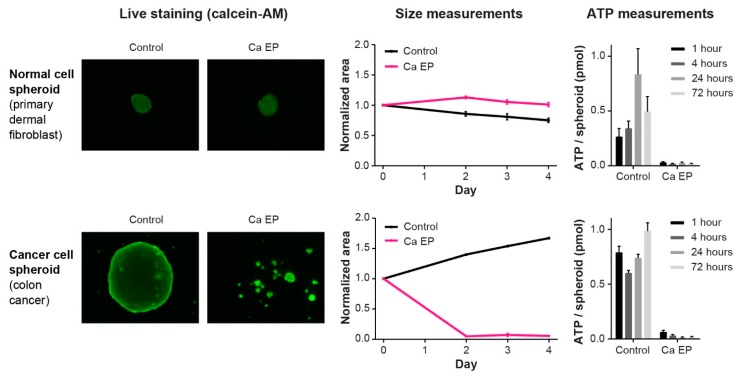
Normal and cancer cell response to calcium electroporation. Calcium electroporation induces different response in normal cell spheroids and cancer cell spheroids where cancer cell spheroids decrease in size while no change in size is seen in the normal cell spheroids. However, intracellular ATP level is depleted in both normal and cancer cell spheroids for up to 72 hours, which the normal cells are able to survive. Adapted from Frandsen, Gibot et al., 2015 [85].

When testing a potential novel anti-cancer treatment it is of the upmost importance to investigate the effect on the surrounding normal tissues. For calcium electroporation, the effect on six normal cell lines has been investigated in vitro as well as the effect on normal skin- and muscle tissue on mice (Table 1). The difference in effect of calcium electroporation between normal and malignant muscle cell lines has been compared in two in vitro studies [78,82]. This showed significantly less cell death in normal muscle cells compared with the cancer muscle cells when treating with calcium electroporation using low concentrations of calcium (0.5 mM) and with high concentrations of calcium (5 mM calcium) for most of the tested electroporation parameters [78,82]. This was the case for both attached cells and cells in suspension. Thus, normal cells seem less sensitive to calcium electroporation than cancer cells, as seen with electrochemotherapy [86]. When treating normal human umbilical vein endothelial cells, Chinese hamster ovary cells, and human primary dermal fibroblasts in vitro in suspension, however, the cells were highly affected by calcium electroporation [80,81]. When primary normal fibroblasts were treated as spheroids, interestingly no decreased survival was seen [85]. The study clearly showed that calcium electroporation and bleomycin electroporation induced cell death in all three cancer cell spheroids (breast, bladder, and colon cancer) but not in the normal cell spheroid, although ATP depletion was observed in all calcium electroporated spheroids (Figure 3) [85]. These results were confirmed in vivo on mice where the normal tissue surrounding tumors treated with calcium electroporation was far less affected than the tumor tissue [61].

Like electrochemotherapy [87,88], calcium electroporation has been shown to have an anti-vascular effect in vitro and in vivo on both normal and tumor blood vessels [81]. Using a dorsal window chamber tumor model in vivo, it was observed that calcium electroporation disrupted both tumor and normal vessels. Furthermore, calcium electroporation inhibited the ability of endothelial cells to migrate and form capillary-like structures in vitro. The anti-vascular effect, even though it also affects normal blood vessels in the treated area, might be an important contributor to the effects induced by calcium electroporation due to drug entrapment in the treated area.

### 5.3. Mechanisms of Action—Cellular Effects

**Figure 4:**
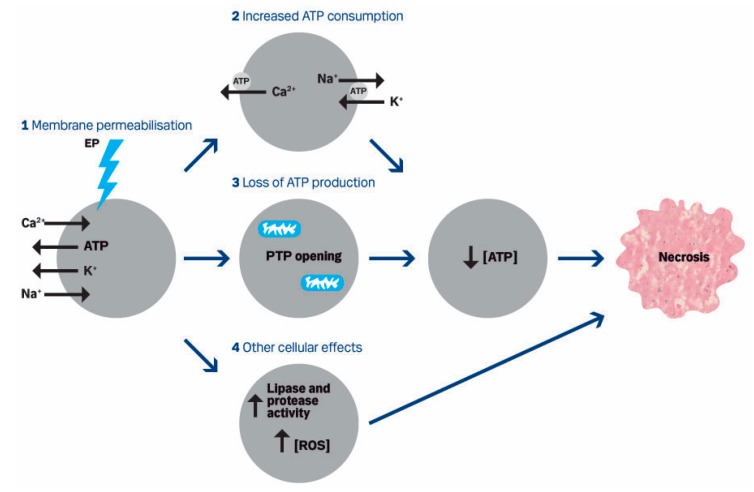
Proposed mechanism of action published in 2012. Calcium electroporation of cells induces influx of calcium and sodium and loss of ATP and potassium due to the concentration gradients (**1**). This may lead to increased ATP consumption (**2**) by the calcium ATPase and sodium potassium ATPase trying to re-establish the calcium homeostasis as well as loss of ATP production (**3**) since increased intracellular calcium can induce the opening of permeability transition pores (PTP) in the mitochondria membrane dissipating the proton gradient. This leads to ATP depletion which has been observed after calcium electroporation as well as necrotic cell death. Other cellular effects (**4**) such as increased activity of lipases and proteases as well as increased concentration of reactive oxygen species (ROS) may also be involved. From Frandsen et al., 2012 [18].

In the first publication about calcium electroporation as an anti-cancer treatment [18], a mechanism of action was suggested (Figure 4), which, in following publications, has been further investigated and supported. Several mechanisms may contribute to the tumor cell kill observed, as well as sparing normal tissue. 

#### 5.3.1. Cell Death

The cell death mechanism induced by calcium electroporation was shown to be apoptosis by Tunnel-assay, Annexin-V staining and transmission electron microscope observations in two in vitro studies [78,82]. In three other studies, it was shown to be necrosis by cell morphology observations and flow cytometry in vitro [81] and in vivo by histological analyses [18,61]. Similar results have been observed with nanosecond pulsed electric fields (nsPEF), an electroporation method using shorter, higher voltage pulses (e.g., in the range of 3–600 ns and 3–40 kV/cm) than classical reversible electroporation, that also induces uptake of extracellular calcium. The studies showed that nsPEF induce apoptosis and necrosis facilitated by calcium uptake depending on cell type [89,90]. Calcium electroporation might also induce both types of cell death depending e.g., on cell type, calcium concentration, time of measurement, and method to determine cell death. 

As described, calcium can pass through the plasma membrane after application of nsPEF, but the pulses will also pass the cell membrane due to the relatively long relaxation time, and thereby also affect intracellular organelles such as mitochondria and ER. A rise of the intracellular calcium concentration is induced through entry of calcium from the extracellular space as well as release from ER, which induces cell death [91,92,93]. This method has been tested for treatment of tumors in vivo [94] and in the clinic [95] and the effect of the treatment may be enhanced by increased extracellular calcium [89,90]. Calcium in combination with other permeabilisation methods has also successfully induced cell death. Irreversible electroporation is a method where very high electric fields and a higher number of pulses are used, resulting in irreversible permeabilisation of the plasma membrane. This method has been studied in vitro, in vivo, and in patients for treatment of tumors, without addition of drugs [96,97,98]; however, recent studies show enhanced efficacy of irreversible electroporation when combined with calcium [99,100,101,102]. Calcium has been used to eliminate tumors when combined with another permeabilisation method, sonoporation [79].

#### 5.3.2. Calcium Uptake

Classical reversible electroporation pulses induce permeabilisation of the cell membrane, and a recent study shows also permeabilisation of internal membranes. Thus cytosolic calcium might increase due to influx of extracellular calcium and from the intracellular calcium stores, such as ER [103]. In vitro studies have shown that intracellular calcium content increases in both cancer cells and normal cells after treatment with calcium electroporation, using different reversible electroporation parameters [61,82,104,105]. This has been confirmed in vivo where calcium content increased significantly in tumor and skin tissue after calcium electroporation; thus, other factors may contribute to the difference in sensitivity between cancer cells and normal cells [61]. Interestingly, in murine muscle tissue, calcium content did not increase significantly after calcium electroporation, which could be due to the effective maintenance of muscle calcium homeostasis in general and could explain the lower sensitivity to calcium electroporation in muscle cells and tissue. Since the intracellular calcium content is similar after treatment, the difference in sensitivity between normal skin and cancer tissue might be explained by differences in the intracellular management of calcium. It has been indicated that membrane repair is more effective in normal cells compared with cancer cells [57], which could indicate that the normal cells can begin to re-establish the ion homeostasis earlier than the cancer cells. A similar effect has been shown using nsPEF where cancer cells were more permeabilized and less viable than normal cells [106]. Furthermore, a recent study indicated that the membrane composition, shown by the phase transition temperature and lipid composition, might contribute to differences in sensitivity to calcium electroporation between cell lines, which could be due to differences in the degree and/or duration of the permeabilisation [83]. 

#### 5.3.3. Calcium Transporters

With possible differences in the degree of permeabilisation and/or membrane repair, re-establishment of the intracellular calcium level after treatment with calcium electroporation may differ between cell types. This has been investigated in vivo [61]. As described, total intracellular calcium content after calcium electroporation clearly increased in tumors as well as in normal surrounding skin shortly after treatment. Interestingly, the calcium content decreased to a level close to untreated controls 4 hours after treatment in normal skin tissue, indicating that normal cells of the skin are better at extruding calcium than tumor cells [61]. As explained in Figure 2, calcium is extruded from cells by the Na^+^/Ca^2+^-exchanger (NCX), Na^+^/Ca^2+^/K^+^-exchanger (NCKX), and plasma membrane calcium ATPase (PMCA). The expression of NCX has been determined in untreated normal and malignant muscle cells showing a higher expression in differentiated normal cells than differentiated malignant cells (no difference in undifferentiated cells) [82]. Moreover, after calcium electroporation the expression of NCX increased in normal muscle cells but decreased in malignant muscle cells, indicating that normal cells can extrude more calcium than cancer cells [82]. This was also supported when investigating the PMCA expression level [61,82]. This showed a significantly higher expression of total PMCA and of PMCA isoform 4 in the two tested normal cell lines compared with the five tested cancer cell lines [61,82]. A significantly higher expression of PMCA isoform 3 was also observed in the malignant muscle cells compared with the normal muscle cells [82]. No significant difference was observed in the expression of PMCA isoform 1 in the tested cell lines [61,82]. PMCA is encoded by four genes (PMCA1–4) with many different splice variants [2,4,28,107] where PMCA1 and 4 are expressed in all cells. The dissociation constant (Kd) for ATP for PMCA1 is lower than the Kd for PMCA4; thus, PMCA1 is regulating the cytosolic calcium concentration at low calcium concentrations whereas PMCA4 pumps calcium out of the cell at higher calcium concentrations [107,108,109]. Since PMCA4 is lower in cancer cells than normal cells, it could indicate that cancer cells might have a reduced ability to decrease the high cytosolic calcium concentrations induced by calcium electroporation. The differences in PMCA level were observed at the protein level; however, no difference was seen on the mRNA level [61], indicating that the difference is caused after mRNA synthesis. 

#### 5.3.4. ATP

As previously described, PMCA is an ATP-dependent calcium pump, and the supposed increased activity of PMCA has an effect on the intracellular ATP level. The intracellular ATP level is depleted for up to 8 hours after calcium electroporation in vitro using increasing calcium concentration and/or increasing electric field [18,77,81]. This is likely due to direct loss of ATP through the permeabilized cell membrane [110,111], increased ATP consumption by ATP-dependent pumps [112] and loss of ATP production due to opening of permeability transition pores in the mitochondria membrane [112] (see Figure 4). Loss of mitochondrial membrane potential has been observed in a recent, unpublished study [113], which supports the proposed mechanism of action. The ATP level is also decreased in spheroids and in tumors in vivo after treatment with calcium electroporation, and the decrease is similar in normal and cancer cells [61,85]. This indicates that both normal and cancer cells try to re-establish the intracellular calcium concentration; however, normal cells are more resilient to ATP depletion. Due to the decreased ATP level after calcium electroporation, it has been speculated that the effect of calcium electroporation together with metformin could be synergistic. The anti-diabetic drug metformin reduces intracellular ATP level by mild and specific inhibition of the mitochondrial respiratory chain complex I [114]. However, when testing the effect of metformin together with calcium electroporation on bladder, breast, colon, and small cell lung cancer tumors in vivo, no increased effect on ATP level, tumor size, nor survival was seen compared with calcium electroporation alone [80].

#### 5.3.5. Other Cellular Effects

Expression of the intracellular calcium channel, ryanodine receptor (RyR), has also been investigated in malignant and normal muscle cells in vitro, showing decreased expression after calcium electroporation in the differentiated cell lines, both normal and malignant, indicating less calcium-induced calcium release from the sarcoplasmic reticulum in the muscle cells [82]. Thus, the expression of this calcium channel seems not to contribute to the difference in effect of calcium electroporation between normal and malignant muscle cells. In the same study, the cytoskeleton structure was also investigated before and after calcium electroporation. The amount of F-actin filaments did not change after treatment with calcium electroporation, but were better organized in the normal muscle cells than the malignant muscle cells. Moreover, expression of the zyxin protein, which is involved in modulating cytoskeleton organization, increased in normal cells while it decreased in malignant cells after calcium electroporation [82]. Investigations of the cellular ultrastructure of normal and malignant muscle cells by transmission electron microscopy have shown that calcium electroporation affects the cellular structure more in malignant muscle cells than normal muscle cells (e.g. increased variety of secretory vesicles and swollen mitochondria) [82], which might also affect survival of the cells after calcium electroporation. Although calcium electroporation has several intracellular effects, the treatment does not induce any genotoxic effects [113], which is important for the normal surviving cells.

In the proposed mechanism of action (Figure 4), other cellular effects such as increased level of reactive oxygen species (ROS) are suggested. A study with nsPEF showed that the increased intracellular calcium level induced by nsPEF stimulates ROS generation, indicating that calcium electroporation using classical reversible electroporation might also stimulate ROS generation. 

Altogether, these studies show how calcium electroporation affects cellular calcium homeostasis, energy metabolism and structure that may all contribute to the difference in sensitivity between normal and malignant cells, but further studies are needed to fully elucidate the mechanism behind calcium electroporation.

### 5.4. Mechanisms of Action—Immune Response

**Figure 5:**
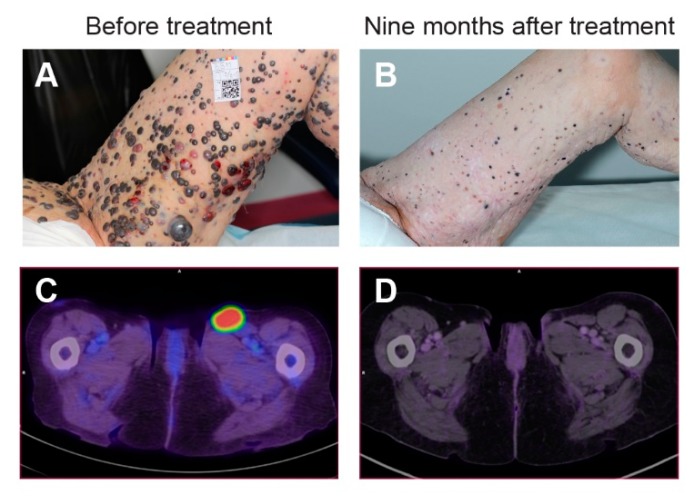
Systemic immune response after calcium electroporation. A patient with cutaneous metastases from malignant melanoma was treated with electrochemotherapy using intravenous bleomycin. Four months after treatment of some of the multiple metastases, new metastases appeared. Two of these were treated (**A**), one with calcium electroporation and one with electrochemotherapy using intratumoral bleomycin, both with complete response within 6 months. Nine months after this treatment complete levelling of all cutaneous metastases appeared (**B**) as well as no or limited signs of previously enlarged lymph nodes on PET/CT scan (**C**,**D**). Adapted from Falk et al., 2018 [19].

Besides the local effects observed in treated cells and tissues, calcium electroporation has also been shown to induce a systemic response. In an in vivo study on immunocompetent mice, calcium electroporation treatment of colon cancer tumors induced a complete response. Interestingly, when the treated mice were re-challenged with the same cancer cells, no new tumors were formed while tumors developed when pre-treated mice were challenged with different cancer cell types [79]. Contrarily, in immunocompromised mice no long-lasting response was seen after treatment with calcium electroporation of the same tumor type. In this study, calcium electroporation in vivo induced an increased systemic release of pro-inflammatory cytokines, and calcium electroporation in vitro induced an increased release of the High Mobility Group Box 1 protein (HMGB1), a damage-associated molecular pattern molecule important in immunogenic cell death [79]. Thus, this study indicates that calcium electroporation activates immune stimulators and can induce a systemic immune response. This has also been indicated in an in vivo study using calcium in combination with irreversible electroporation, which induced an anti-tumor immune response with increased T-cell numbers and less suppressor cells [101]. In the described calcium electroporation in vivo study and in another in vivo study, electrochemotherapy also induced a systemic immune response in mice [79,115]; however, to our knowledge no clinical observations of systemic response or response in untreated lesions after treatment with electrochemotherapy have been published. Interestingly, in the first clinical trial with calcium electroporation, a patient experienced systemic immune response against untreated tumors (Figure 5), which is described Section 5.6.

### 5.5. Veterinary Studies

Calcium electroporation has been tested in veterinary medicine as an alternative to electrochemotherapy or in combination with electrochemotherapy. Two case reports describe treatment of a canine and a feline malignant melanoma with calcium electroporation. In the study with the canine melanoma [116], the tumor was treated with electrochemotherapy and laser surgery causing decrease of the tumor mass. Progression of the metastatic disease was observed two weeks after the treatment resulting in treatment of lymph node metastases with calcium electroporation using a low calcium concentration (5 mM). Thirty days later, complete remission of metastases was observed indicating a systemic immune response. Unfortunately, the dog was euthanized two months after diagnosis due to brain metastases. In the case with a feline melanoma [117], the tumor was treated with electrochemotherapy with good effect but recurrence occurred three months after treatment. Two metastases were treated with electroporation in combination with both bleomycin and a low calcium dose (9 mM), thus electrochemotherapy combined with calcium. This resulted in complete response one month after treatment but with recurrence four months later. Only mild, local side effects were observed. 

Recently, the safety and efficacy of calcium electroporation was investigated in 16 sarcoids on ten horses [118]. The treatment was well tolerated by all horses. The sarcoids were surgically excised 7, 14, and 21 days after treatment for histological analyses, which showed necrosis in 13 of 16 sarcoids. In nine of these the fraction of necrosis was higher than 50%. Furthermore, the histological analyses showed hemorrhages, ulceration, thrombosis, and calcification in most of the treated sarcoids. Also in this study, surrounding normal tissue seemed to be less affected by the treatment than the tumor [118].

### 5.6. Clinical Trials

The first human clinical trial with calcium electroporation was published in 2017 [19]. Since calcium chloride is an approved drug for clinical use and electroporation is an established clinical method, a clinical trial with calcium electroporation could be readily initiated after the first preclinical studies. The randomized, double-blinded phase II trial compared tumor response to calcium electroporation and electrochemotherapy with bleomycin, with a non-inferiority margin of 15%. Seven patients with cutaneous metastases from breast cancer (six patients) and malignant melanoma (one patient) were included in the trial. The patients had 47 metastases in total, of which 37 were randomized and evaluated for response and 10 metastases were treated and used for biopsies. The metastases on each patient were randomized to either calcium electroporation or electrochemotherapy and since both calcium and bleomycin are clear liquids, the trial could be double-blinded. Calcium (220 mM) and bleomycin (1000 IU/mL) were injected intratumorally in a volume equivalent to 50% of the tumor volume for tumors above 0.5 cm^3^ and in a volume equivalent to the tumor volume for smaller tumors (<0.5 cm^3^) [19]. A slightly higher calcium concentration was used in this clinical trial than in the murine studies in order to improve the chance of efficacy in this first-in-man trial. Immediately after injection, electric pulses were delivered to the tumor as described in the standard operating procedure for electrochemotherapy [65], which has been shown to be effective for calcium electroporation in vitro and in vivo [61,84]. The study showed that calcium electroporation is a safe and effective treatment of the included cutaneous metastases (< 3 cm in diameter) where the objective tumor response six months after treatment was 72% for calcium electroporation (with 66% complete response) and 84% for electrochemotherapy (with 68% complete response) [19]. In this small study, there was no significant difference in objective response between the two treatments and the study showed non-inferiority for calcium electroporation. A significant difference in objective response was observed between previously irradiated and non-irradiated metastases, respectively 46% and 81%, which might be due to difficulties inserting the needle electrodes and poorer drug distribution in fibrotic tissue [19]. Only minimal adverse events were reported for both treatments, including ulcers in the treated area. Interestingly, ulcers only appeared in the tumor region, indicating that the normal surrounding skin was spared [19] as observed in the preclinical studies [61,85]. 

Interestingly, the patient with malignant melanoma in this clinical trial experienced a systemic response [119]. The patient had widespread cutaneous metastases from malignant melanoma (see Figure 5) where more than 100 of the metastases were treated with electrochemotherapy using intravenous bleomycin according to the standard operating procedures [65]. Four months after the treatment new metastases appeared. Two of these metastases were treated in the clinical trial described above [19], one with calcium electroporation and one with electrochemotherapy, both with complete response 6 months after treatment [119]. Interestingly, nine months after the treatment with calcium electroporation and electrochemotherapy complete levelling of all cutaneous metastases appeared, also of untreated metastases, and biopsies three months later showed no sign of malignant melanoma [119]. At this time, two lymph nodes that were pathologically enlarged before the treatments showed no or minimal activity on the PET/CT scan. This indicates that the treatment with electrochemotherapy and calcium electroporation induced a systemic immune response (Figure 5) [119]. 

After the first clinical trial using calcium electroporation, several studies commenced (Table 2). A phase I study treating recurrent mucosal head and neck cancer patients with calcium electroporation has recently been published [120]. Six patients that were previously irradiated, deemed inoperable, and without further treatment options, were included in the trial testing safety of the treatment on larger mucosal head and neck cancers. The procedures were performed without complications. The calcium level in serum was measured 30 min and 6 h after treatment showing no sign of hypercalcemia after the treatment. The treatment was well tolerated with three partial responses two months after treatment. One patient experienced complete response later on and was without clinical evidence of disease 12 months after treatment. 

A confirmatory trial based on the same protocol as the first clinical trial [19] has included 7 patients with cutaneous metastases from malignant melanoma (six patients) and breast cancer (one patient) [121]. Similar calcium concentrations were used, however both linear and hexagonal electrodes were used in this study (only linear in the first clinical study). The study confirmed that calcium electroporation is a safe and feasible treatment for small cutaneous metastases and the objective tumor response six months after treatment was 33% for calcium electroporation (with 22% complete response) and 53% for electrochemotherapy (with 40% complete response). Although, response rates in both treatment groups are lower than in the first clinical study, calcium electroporation was also in this study non-inferior to electrochemotherapy.

A larger, multicenter study on cutaneous tumors investigating the response rate after calcium electroporation will commence in 2020 (clinicaltrials.gov #NCT04225767).

Furthermore, two clinical trials with calcium electroporation of colorectal cancer are ongoing. In one of these, six patients with inoperable colorectal cancer will be included for treatment with calcium electroporation to evaluate safety and efficacy of the treatment (clinicaltrials.gov #NCT03542214). The other ongoing study with colorectal cancer will include 24 patients with rectal cancer or left-sided colon cancer that will be treated with calcium electroporation two weeks prior to intended curative surgical removal of the tumor. The primary outcome of this study is safety and the secondary outcome is evaluation of tumor immunological changes (clinicaltrials.gov #NCT03694080), with perspective hope of reducing micrometastases after surgery and lower recurrence rate. Finally, a trial on keloids (clinicaltrials.gov #NCT01941914), proliferative scars, has completed the inclusion of 7 patients and the manuscript is in preparation (J. Gehl).

## 6. Cost and Feasibility of Intervention

In 2015, cancer was responsible for around 8.8 million deaths globally and approximately 70% of cancer-related deaths occurred in low- and middle-income countries [122]. At the same time, drug prices keep rising and the economic impact of cancer is increasing, with the global annual economic cost of cancer estimated at approximately US$ 1.16 trillion in 2010 [122,123]. There is therefore a great need of new cancer treatments to lower the cost of treatments in high-income countries [124] as well as in low- and middle-income countries [123,125,126]. Calcium electroporation is a novel treatment that is efficient and inexpensive. Furthermore, calcium electroporation is feasible also in low- and middle-income countries since the treatment is a once-only treatment that can be used both in hospitals and in smaller mobile outreach clinics. Calcium electroporation treatment requires calcium, an electric generator and electrodes. Calcium is inexpensive, easily available, heat-stable, and has a long shelf life making it practical for most clinical facilities. Electric generators and electrodes approved for clinical use are available and more affordable generators may be produced depending on the certification cost, a one-time cost that depends on the country in question. Affordable electrodes may also be produced. Altogether, calcium electroporation is an efficient and affordable treatment that can be introduced into most clinical settings in high-, middle-, and low-income countries.

## 7. Perspectives

Calcium electroporation has been proven safe and efficient in the first clinical trials. It is a local treatment limited by the access to the tumor for calcium injection and electrode placement. 

Since calcium electroporation does not include chemotherapeutic drugs, it can be used, not only by oncologists, but also by e.g. interventional radiologists and surgeons. Likewise, veterinarians are interested in calcium electroporation to increase safety for the personnel, reduce price and avoid (expensive) handling of biological hazard waste, which may be required in the first days after treatment with a chemotherapeutic drug.

Initial clinical studies have used a high dose of calcium (220–225 mM in a volume equivalent to 50% of the tumor volume) but data also points to lower doses of calcium being effective [61,116]. This needs to be further investigated; nonetheless, for a safety point of view high doses of calcium were well tolerated [19,120].

In the clinical studies described in this review, the European Standard Operating Procedure on Electrochemotherapy (ESOPE) was followed; however, it has been shown that ESOPE-equivalent or comparable pulsing protocols can be used with calcium electroporation with similar effect [104,105,127]. Furthermore, calcium may also enhance the effect of treatments using irreversible electroporation [99,100,101,102], nanosecond pulse electric fields (nsPEF) [89,90] and sonoporation [79]. 

The intriguing observation that anti-tumor immune response may be elicited by calcium electroporation warrants further investigation. Indeed, a clinical study is now looking at calcium electroporation in a neoadjuvant setting for colorectal cancer and coming preclinical and clinical studies may investigate possible immune response activation by combining calcium electroporation with immune stimulating agent or by performing repeated treatments.

## 8. Conclusions

Calcium electroporation is a novel, efficient, safe and inexpensive anti-cancer treatment. The first clinical trials using calcium electroporation showed safety and efficacy of the treatment of cutaneous metastases and recurrent head and neck cancer, and more clinical trials are recently completed or ongoing. Interestingly, there has not only been an evolution in terms of use of calcium electroporation for new tumor histologies, but also in terms of indication since the treatment was initially investigated in patients without further treatment options and now is also being investigated as a neoadjuvant treatment prior to intended curative surgery.

Most preclinical studies show a difference in sensitivity between normal and malignant cells and tissues, thus sparing normal surrounding tissue when treating tumors with calcium electroporation, which has also been indicated in a veterinary study and the first clinical trial. This difference in sensitivity may be explained, in part, by differences in membrane repair after electroporation, differences in the expression of calcium transporters, and changes in cellular structures. Further studies investigating the mechanism of action are evolving.

Calcium electroporation is a local treatment though systemic immune effects have been indicated in vivo where calcium electroporation induced a systemic immune response with increased systemic release of pro-inflammatory cytokines. Furthermore, a systemic immune response was observed in a dog treated with calcium electroporation and, in the first clinical trial, a patient experienced a systemic response. Calcium electroporation, has been studied in vitro, in vivo, in veterinary studies and in clinical trials as a novel anti-cancer treatment. The results of these studies have shown a safe and efficient treatment that may induce a systemic response, yet further investigation is needed to fully elucidate the potential of this novel treatment.

With increasing cancer incidence and treatment expenses worldwide, efficient and inexpensive anti-cancer treatments are warranted in low- and middle-income countries as well as high-income countries. As an efficient once-only treatment that can be used both in hospitals and in smaller mobile outreach clinics, calcium electroporation may benefit patients throughout the world by introducing an extra tool in the armamentarium of cancer treatment.

## Figures and Tables

**Table 1:** Calcium electroporation of normal vs. malignant cells. Note the difference between in vitro and in vivo observations.

Model	Author	Investigated Cell Types	Cell Condition	Observation
In Vitro	Zielichowska et al. 2016 [78]	Murine normal muscle cells; murine sarcoma cells	Suspension	Less cell death in normal cells than malignant cells
	Szewczyk et al. 2017 [82]	Murine normal muscle cells; murine sarcoma cells	Suspension and attached (differentiated and undifferentiated)	Less cell death in normal cells than malignant cells
	Frandsen et al. 2018 [80]	Human primary dermal fibroblasts	Suspension	Induced cell death in normal cells
	Staresinic et al. 2018 [81]	Human umbilical endothelial cells; Chinese hamster ovary cells	Suspension	Induced cell death in normal cells
3D Spheroid	Frandsen et al. 2015 [85]	Human breast-, bladder-, and colon cancer and primary dermal fibroblasts	Spheroids	Cell death induced in all three cancer cell lines but affected normal cells less
In Vivo	Frandsen et al. 2017 [61]	Human SCLC *; breast-; bladder-; colon cancer tumors; normal skin and normal muscle	Tissue	Induced necrosis in all tumor types but limited effect on normal tissue

* Small cell lung cancer (SCLC).

**Table 2:** Clinical trials with calcium electroporation.

Clinicaltrials.gov	Condition	Palliative/Neoadjuvant	Published/Ongoing
NCT01941901	Cutaneous metastases from breast cancer and malignant melanoma	Palliative	Falk et al., 2018 [19]
NCT03628417	Cutaneous metastases from breast cancer and malignant melanoma	Palliative	Ágoston et al, 2020 [121]
NCT04225767	Cutaneous tumors	Palliative	Ready to start accrual Feb. 2020
NCT03051269	Recurrent head and neck cancer	Palliative	Plaschke et al., 2019 [120]
NCT03542214	Colorectal cancer	Palliative	Ongoing
NCT03694080	Colorectal cancer	Neoadjuvant	Ongoing
NCT01941914	Keloid	--	Inclusion completed (7 patients)
